# Use of uterine manipulator in laparoscopic colorectal surgery

**DOI:** 10.4103/0972-9941.72602

**Published:** 2010

**Authors:** P R Shah, J Rogers, S Chawathe, P N Haray

**Affiliations:** Department of Surgery, Prince Charles Hospital, Merthyr Tydfil, UK

**Keywords:** Uterine manipulator, colorectal surgery

## Abstract

Laparoscopic colorectal surgery has become more common with the increase in the number of trained surgeons. We have used a disposable uterine manipulator to retract the uterus. This technique has been found to be very useful for laparoscopic low anterior resection and abdomino-perineal resection in females.

## TECHNICAL TIP

Laparoscopic colorectal surgery is becoming more common and more surgeons are being trained. It poses various challenges e.g. mobilising the splenic flexure, dividing the gastro-colic omentum or performing total mesorectal excision. While performing rectal dissection in females, the uterus, the tubes and ovaries can flop down making anterior and lateral dissection very difficult, time consuming and occasionally frustrating. There is then usually need to either suspend the uterus with sutures from the anterior abdominal wall or use a specific retractor via one of the laparoscopic ports.

**Figure 1:**
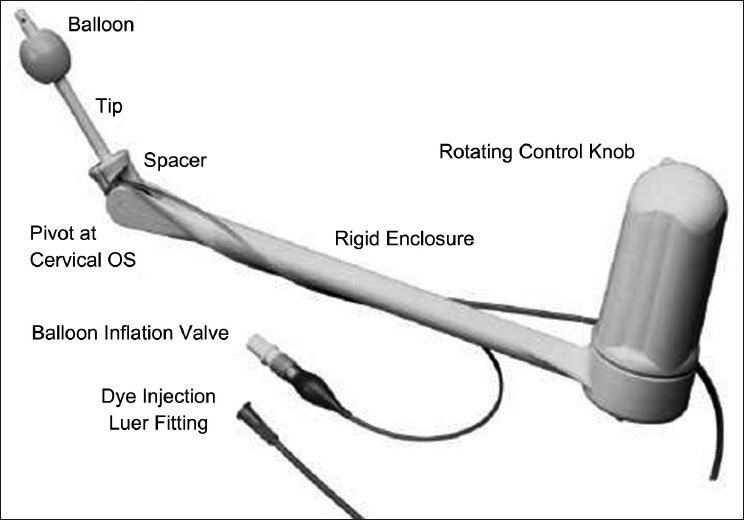
ClearView™ - Disposable uterine manipulator

We have used the ClearView™ - Disposable uterine manipulator (Clinical Innovations LLC, Murray, UT, USA) [Figure 1] to retract the uterus. It provides full anteversion, retroversion and lateral motion of uterus. The ClearView™ allows for easy access and simultaneous use with laparoscope without breaking the sterile barrier. It is easy to use and once deployed, can be locked in the position required and used as a self-retaining retractor. With the ClearView™, no assistant is needed to hold the manipulator. It also alleviates the need to use one of the ports for uterine retraction. We use this technique routinely for laparoscopic low anterior resection and abdomino-perineal resection in females.

